# Naringenin is a Potential Anabolic Treatment for Bone Loss by Modulating Osteogenesis, Osteoclastogenesis, and Macrophage Polarization

**DOI:** 10.3389/fphar.2022.872188

**Published:** 2022-05-02

**Authors:** Xin Zhou, Zheng Zhang, Weiwei Jiang, Miao Hu, Yichen Meng, Wenfang Li, Xuhui Zhou, Ce Wang

**Affiliations:** ^1^ Department of Orthopedics, Changzheng Hospital, Second Military Medical University (Naval Medical University), Shanghai, China; ^2^ College of Basic Medicine, Second Military Medical University (Naval Medical University), Shanghai, China; ^3^ Department of Critical Care Medicine, Changzheng Hospital, Second Military Medical University (Naval Medical University), Shanghai, China

**Keywords:** naringenin, osteoblast, osteoclast, macrophage polarization, bone loss

## Abstract

Bone undergoes constant remodeling of formation by osteoblasts and resorption by osteoclasts. In particular, macrophages have been reported to play an essential role in the regulation of bone homeostasis and regeneration. Naringenin, the predominant flavanone in citrus fruits, is reported to exert anti-inflammatory, anti-osteoclastic, and osteogenic effects. However, whether naringenin could modulate the crosstalk between macrophages and osteoblasts/osteoclasts remains to be investigated. In this study, we confirmed that naringenin enhanced osteogenesis and inhibited osteoclastogenesis directly. Naringenin promoted M2 transition and the secretion of osteogenic cytokines including IL-4, IL-10, BMP2, and TGF-β, while suppressing LPS-induced M1 polarization and the production of proinflammatory factors such as TNF-α and IL-1β. In addition, the coculture of primary bone mesenchymal stem cells (BMSCs)/bone marrow monocytes (BMMs) with macrophages showed that the naringenin-treated medium significantly enhanced osteogenic differentiation and impeded osteoclastic differentiation in both inflammatory and non-inflammatory environment. Moreover, *in vivo* experiments demonstrated that naringenin remarkably reversed LPS-induced bone loss and assisted the healing of calvarial defect. Taken together, naringenin serves as a potential anabolic treatment for pathological bone loss.

## Introduction

Bone undergoes constant remodeling mediated by osteoblasts and osteoclasts (Chen et al., 2020). Deficient bone formation by osteoblasts and/or excessive bone resorption by osteoclasts lead to osteopenic diseases including osteoporosis and delayed healing of bone defect (Schindeler et al., 2008). It was estimated that more than 200 million individuals suffered from low bone density worldwide, which led to over 8 million fractures every year, causing relative healthcare cost of multi-billion dollars (Sözen et al., 2017; Muñoz et al., 2020). Anti-resorptive drugs including bisphosphonates and denosumab have been used against pathological bone loss, but they are far from ideal for the side effects of atypical fractures and osteonecrosis of the jaw (Reid, 2020). In addition, these medications do not restore the impaired osteogenesis and bone formation. Therefore, it is important to develop therapeutic agents for bone loss by not only inhibiting bone resorption but also promoting bone formation.

Immune cells are important constituents of bone remodeling microenvironment, in which macrophages have been reported to play an essential role in bone homeostasis and regeneration (Pajarinen et al., 2019; Sun et al., 2021). The plasticity and heterogenicity of macrophages in response to various environments has been conceptualized as polarization (Martinez et al., 2008). Generally, macrophages are polarized into two subgroups: proinflammatory M1 phenotype and anti-inflammatory M2 phenotype (Martinez et al., 2008). M1 macrophages with CCR7 and CD11c markers induce bone marrow inflammation by secreting cytokines such as TNF-α and IL-1β which activate excessive osteoclastogenesis in cases of aging, infection, and early stages of bone fracture (Pajarinen et al., 2019; Yang and Yang, 2019). M2 macrophages with CD163 and CD206 markers release factors including IL-4, IL-10, BMP2, and TGF-β to maintain normal bone marrow environment and promote osteogenesis, as well as angiogenesis in the later stages of fracture healing (Pajarinen et al., 2019; Yang and Yang, 2019). Prolonged activity of M1 macrophages and high M1/M2 ratios contribute to chronic inflammatory states, causing bone mass destruction and undesirable prognosis for bone fractures (Li et al., 2021). Therefore, targeting M1-M2 transition is a promising strategy for rebuilding the balance of bone formation and resorption.

Naringenin, belonging to the flavanone family, is rich in citrus fruits and reported to exert various pharmacological effects (Miles and Calder, 2021). Due to its anti-inflammatory activities, naringenin is considered as a potential therapeutic agent in treating multiple inflammation related diseases including sepsis, fibrosis, atherogenesis, and cancer (Zeng et al., 2018). For example, naringenin suppressed NF-κB and MAPK activation via AMPK-ATF3 signaling in macrophages to attenuate murine endotoxemia (Liu et al., 2016). *In vitro* experiments also showed naringenin treatment inhibited proinflammatory cytokine levels in macrophages by inhibiting NF-κB activation (Yang et al., 2021). In addition, some pioneer studies illustrated the beneficial effects of naringenin on bone diseases such as chronic arthritis and osteoporosis (Kaczmarczyk-Sedlak et al., 2016; Manchope et al., 2018). Naringenin could promote osteogenic differentiation of human periodontal ligament stem cells (hPDLSCs) and suppress osteoclastogenesis through enhancement of helper T-cell-secreted IL-4 (Wang et al., 2018; Zhang L. et al., 2021; Wang et al., 2021). However, most of these studies do not supply high-quality data of naringenin’s effects *in vivo*. Also, how naringenin modulates the crosstalk between macrophage and bone remodeling in the field of osteoimmunology remains to be explored.

In this article, we studied the influence of naringenin on osteogenesis, osteoclastogenesis, and macrophage polarization. The crosstalk between macrophages and osteoblasts/osteoclasts in response to naringenin in non-inflammatory and inflammatory conditions was investigated by the coculture system. In addition, mice models of LPS-induced bone loss and calvarial defect were used to further demonstrate the therapeutic potential of naringenin on bone diseases *in vivo*.

## Materials and Methods

### Reagents

Naringenin (BioChemParter, China; BCP31780; purity 98%); dimethyl sulfoxide (DMSO, Yeasen, China; 60313ES60); lipopolysaccharide (LPS, Beyotime, China; S1732); Dulbecco’s modified eagle medium (DMEM, Cytiva, China; SH30021.01); Minimal essential medium alpha modification (α-MEM, Cytiva, China; SH30265.01); fetal bovine serum (FBS, gibco, United States; 10099141); penicillin/streptomycin solution (Yuanye, China; R20016);; ACK lysing buffer (Thermo Fisher scientific, United States; A1049201); β-glycerophosphate (sigma, United States; G9422); L-ascorbic acid (sigma, United States; A4403); dexamethasone (MKBio, China; MX3252); BCIP/NBT Alkaline Phosphatase Color Development Kit (Beyotime, China; C3206); Alizarin Red S (sigma, United States; A5533); M-CSF (Novoprotein, China; CB34); RANKL (Novoprotein, China; CR06); Acid Phosphatase, Leukocyte (TRAP) Kit (Sigma, United States; 387A); TRITC Phalloidin (Solarbio, China; CA1610); CCK8 kit (Dojindo, Japan; CK04); RNA extracting kit (Yishan, China; RN001); PrimeScript™ RT Master Mix (Takara, Japan; RR036); TB Green^®^ Premix Ex Taq™ (Takara, Japan; RR420); anti-RUNX2 rabbit pAb (Servicebio, China; GB13264); anti-osteocalcin (Bglap) rabbit mAb (Abclonal, China; A20800); anti-CTSK rabbit pAb (Abclonal, China; A1782); Anti-DCSTAMP Antibody, clone 1A2 (EMD Milipore, Sigma, United States; MABF39-I); anti-osteocalcin polyclonal antibody (Proteintech, United States; 16157-1-AP); PE anti-mouse CD197 (CCR7) antibody (BioLegend, United States; 120105); APC anti-mouse CD206 (MMR) antibody (BioLegend, United States; 141707); osteocalcin ELISA Kit (J&L Biological, China; JL20366); and CTX-I ELISA Kit (J&L Biological, China; JL20123).

### Preparation of Naringenin Solution

Naringenin was dissolved in DMSO at a concentration of 80 mM as stock solution. When the naringenin stock solution was used, it was diluted in the culture medium or physiological saline at appropriate concentration and then water bathed at 37°C until totally dissolved. A corresponding concentration of DMSO was used for the control group.

### Isolation of Primary BMSCs and BMMs

Isolation and culture of BMSCs (osteoblast progenitors) and BMMs (osteoclast progenitors) from mice were reported previously (Maridas et al., 2018). Briefly, euthanize the mice, harvest femurs as well as tibias and make small cuts at both ends of the bones. Then use sterile syringes to blow the bone marrow into 10-cm dishes with normal culture medium (consisting of α-MEM with 10% FBS and 1% Penicillin/Streptomycin). After 24 h of mixed culture, remove the culture medium, collect non-adherent cells (mainly derived from hematopoietic lineage cells), plate cells after ACK lysis for 5 min and then begin the induction of osteoclast differentiation. Adherent cells (BMSCs) are cultured until it reaches the confluence of 70–80% and then perform osteogenic differentiation induction.

### Osteogenic and Osteoclastogenic Differentiation Assay

The induction of osteogenic differentiation was described previously (Zhang et al., 2021a). Osteogenic medium was prepared by adding 10 mM β-glycerophosphate, 50 μg/ml ascorbic acid, 100 nM dexamethasone, 10% FBS, and 1% penicillin/streptomycin to α-MEM. Primary mouse BMSCs were digested and seeded in 24-well or 12-well plates and cultured in normal medium until they reached the confluence of 60–70%. Then the cells were cultured in osteogenic medium with different concentrations of naringenin (0, 5, 10, 15 μM) and the medium was changed every 2 days. After culturing for 7 days, the cells were harvested for the isolation and purification of total RNA or protein. In addition, Alizarin Red S (ARS) (sigma, United States) and alkaline phosphatase (ALP) staining (Beyotime, China) were performed to evaluate the osteogenesis when BMSCs were cultured for 14 days according to the manufacturer’s instructions.

The protocol for osteoclastogenic differentiation was reported previously (Zhang et al., 2021b). Osteoclastogenic medium: α-MEM with 10% FBS, 1% penicillin/streptomycin, 30 ng/ml M-CSF, and 50 ng/ml RANKL. In brief, non-adherent BMMs were seeded in 24-well or 12-well plates and incubated in osteoclastogenic medium with different doses of naringenin (0, 10, 25, 50 μM). After 5 days, we either harvest the cells for RNA/protein isolation, or perform TRAP (Sigma, United States) or F-actin (Solarbio, China) staining according to the manufacturer’s instructions.

### Cell Culture of RAW264.7 Cells

To investigate the effects of naringenin on phenotype switch of macrophages in non-inflammatory environment, RAW 264.7 cells were seeded in 12-well plates and cultured in normal culture medium (DMEM with 10% FBS and 1% penicillin/streptomycin) with PBS or naringenin (5, 10, 15 μM) for 24 h. Then the cells were harvested for flow cytometry/RNA isolation. Alternatively, to study the influence of naringenin on macrophage polarization in inflammatory environment, LPS was added into culture medium at a concentration of 20 ng/ml and incubated with cells for another 6 h and then the cell samples were harvested for further detection.

### Cell Counting Kit-8 Assay

CCK-8 (Dojindo, Japan) was performed according to the manufacturer’s guidance. Briefly, 2,000 cells (primary BMSCs, BMMs or RAW264.7 cells) were seeded into 96-well with different concentrations of naringenin. After 72 h′ incubation, 10 ul of CCK-8 solution was added into the culture medium and cocultured for 4 h. Then, the optical density (OD) value was measured using a spectrophotometer at 450 nm.

### Flow Cytometry

As reported in previous studies, CCR7 was used as marker for M1 macrophages while CD206 as a marker for M2 macrophages (Vasconcelos et al., 2015; Chen et al., 2019; Peng et al., 2019; Kim et al., 2020; Chen et al., 2021). RAW 264.7 cells were digested and washed with PBS. Then, cells were blocked with CD16/32 for 10 min and incubated with PE-conjugated CCR7 or FITC-conjugated CD206 for 1 h at 4°C. Next, cells were washed and resuspended in PBS and analyzed by using a flow cytometer (CyAn ADP Analyzer, Beckman Coulter).

### RNA Preparation and Quantitative Real-Time Polymerase Chain Reaction

The protocols of this part were described previously (Zhang et al., 2021b). Total RNA was prepared using RNA extraction kit (Yishan, China). Complementary DNA was obtained by PrimeScript™ RT Master Mix. And mRNA levels of Runx2, Alp, Ocn, Acp5, CtsK, Dc-stamp, CCR7, CD11c, CD206, CD163, TNF-α, IL-1β, IL-4, IL-10, and Gapdh were assessed by real-time PCR using TB Green^®^ Premix Ex Taq™. Primers used in this study are listed in Supplementary Table S1.

### Western Blot

As previously reported, western blot was used to measure the protein levels (Zhang et al., 2021a). Total proteins of cells were extracted with radioimmunoprecipitation assay (RIPA), electrophoresed in SDS-PAGE gels, transferred to a polyvinylidene fluoride (PVDF) membrane, and blocked with skimmed milk. Then the membranes were incubated with RUNX2 (1:1000), OCN (1:1000), CTSK (1:1000) and DCSTAMP (1:1000) antibodies, and treated with secondary antibodies. Finally, blotted bands were analyzed by enhanced chemiluminescence reagent system.

### Bone Histomorphometry

Bone histomorphometry experiments including hematoxylin and eosin (HE) staining, OCN immunohistochemistry, tartrate-resistant acid phosphatase (TRAP) staining, calcein staining, and Masson staining were carried out and analyzed as previously described (Zhang et al., 2021b).

### Serum Biochemistry

Blood was collected by heart puncture and then centrifuged at 3000 rpm for 15 min to get sera. Serum OCN and CTX was measured using an ELISA kit (J&L Biological, China) according to the manufacturer’s instruction.

### MicroCT (μCT)

Femoral structure was analyzed by high resolution micro-computed tomography (Skyscan 1172, Bruker, Blgium) just as we previously reported. The setting used was 80 kV, 124 mA, and resolution of 8 mm. The following parameters were obtained: bone volume/total volume (BV/TV), bone surface area/total volume (BS/TV), bone mineral density (BMD), trabecular number (Tb.N), trabecular space (Tb.Sp), and trabecular thickness (Tb.Th).

### *In Vitro* Co-Culture Experimental Design

After the RAW 264.7 cells were incubated with or without naringenin (15 μM)/LPS (20 ng/ml), the culture medium was removed and centrifuged at 12,000 g for 20 min. Then the supernatant was mixed with normal medium (α-MEM with FBS and penicillin/streptomycin) at a ratio of 1:2. Then 5 mM β-glycerophosphate, 50 μM ascorbic acid, and 100 nM dexamethasone were added into the mixed medium to obtain conditioned osteogenic medium (Ob-CM). In a similar manner, the mixed medium was supplemented with 30 ng/ml M-CSF and 50 ng/ml RANKL to get conditioned osteoclastogenic medium (Oc-CM).

Primary mouse BMSCs were seeded into 12-well or 24-well plates in normal medium until they reached the density of 70–80% and then cultured in five different culture mediums for 7–14 days. Con group: normal osteogenic medium; blank RAW group: untreated RAW 264.7 Ob-CM; nar + RAW group: naringenin (15 μM) treated RAW 264.7 Ob-CM; LPS + RAW group: LPS (20 ng/ml) treated RAW264.7 Ob-CM; LPS + nar + RAW group: naringenin plus LPS treated RAW264.7 Ob-CM (Supplementary Figure S1A).

Primary mouse BMMs were seeded into 12-well or 24-well plates in normal medium and then cultured in five different culture mediums for 7 days. Con group: normal osteoclastogenic medium; blank RAW group: untreated RAW 264.7 Oc-CM; nar + RAW group: naringenin-treated RAW 264.7 Oc-CM; LPS + RAW group: LPS treated RAW264.7 Oc-CM; LPS + nar + RAW group: naringenin plus LPS treated RAW264.7 Oc-CM (Supplementary Figure S1B).

### *In Vivo* Experimental Design

To further confirm the therapeutic effects of naringenin on bone loss, we chose two animal models: LPS-induced inflammatory bone loss and calvarial critical defect. Male C57BL/6 mice were purchased from Shanghai Jihui Laboratory Animal Care Co. (Shanghai, China) and kept under specific pathogen free (SPF) conditions at Animal Experimental Center of Naval Medical University (SYXK 2017-0004). All procedures by the guidelines of the Ethics Committee on Animal Experiments of the Naval Medical University were abided.

To investigate the effects of naringenin on LPS-induced bone loss, 15 healthy 8 week old male C57BL/6J mice were randomly divided into three groups (*n* = 5 per group): the con group (saline), LPS group (only LPS and saline injected), and LPS + nar group (LPS and 5 mg/kg Naringenin). LPS and naringenin/saline were intraperitoneally injected every other day for 3 weeks. The animals were then euthanized, and the femora and blood were collected.

To study how naringenin affects bone regeneration, the mice model of calvarial critical defect was constructed as previously described. Fifteen healthy 8-week-old male C57BL/6J mice were randomly divided into three groups (*n* = 5 per group): the con group (only bone defect was created, and then, saline was intraperitoneally injected every other day for 4 weeks), CPC group (a scaffold of calcium phosphate cement (CPC) was filled into the defect, and then, saline was intraperitoneally injected every other day for 4 weeks), and CPC + nar group (after CPC was filled into the defect, naringenin (5 mg/kg) was intraperitoneally injected every other day for 4 weeks). Animals were then euthanized and whole skulls were collected.

### Statistical Analysis

Data were expressed as mean ± standard deviation at least 3 times. Multiple groups were compared by one way ANOVA using GraphPad prism 8.0. Differences were considered significant at *p* < 0.05.

## Results

### Naringenin Promotes Osteogenic Differentiation of Bone Marrow Mesenchymal Stem Cells

**FIGURE 1:**
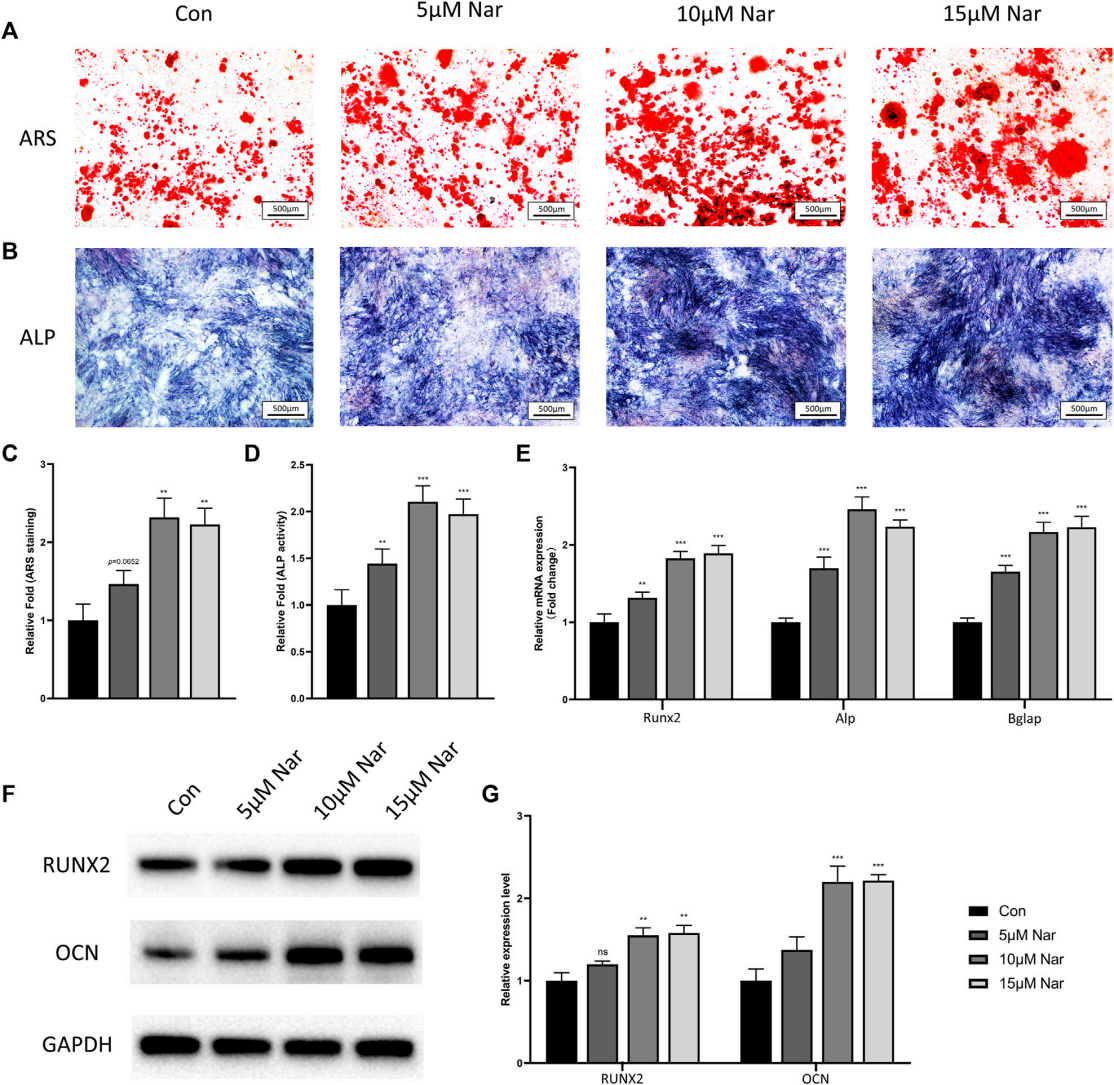
Naringenin promotes osteogenic differentiation of BMSCs. **(A,C)** ARS staining and its quantification. **(B,D)** ALP staining and its quantification. **(E)** Transcript levels of osteoblast markers Runt-related transcription factor 2 (Runx2), alkaline phosphatase (Alp), and bone gamma-carboxyglutamate protein (Bglap). **(F,G)** Western blot and optical density analysis of RUNX2 and OCN with Gapdh as reference. *n* = 4 per group. Data are expressed as mean ± SD. ∗ *p* < 0.05; ∗∗ *p* < 0.01; ∗∗∗ *p* < 0.001. ∗ compared with con group.

To confirm whether naringenin is beneficial for osteogenesis, BMSCs were cultured in osteogenic medium with different concentrations of naringenin. No significant alteration in cell viability and proliferation was shown when BMSCs were treated with naringenin within the dose of 30 μM (Supplementary Figure S2A). As the optimal concentration of naringenin for osteogenic differentiation of hPDLSCs was about 10 μM (Zhang L. et al., 2021), thus we chose 5, 10, and 15 μM naringenin to treat primary mouse BMSCs. The ARS staining showed significant increase in the mineralized nodules and calcium deposition in 10 and 15 μM naringenin-treated group (and a slight increase in 5 μM group, *p* = 0.0652) (Figures 1A,C). In addition, all 5, 10, and 15 μM groups showed a higher ALP activity compared with control group (Figures 1B,D). The mRNA levels of osteogenic genes including Runx2, Alp, and Bglap (gene encoding OCN) were found dramatically elevated under naringenin treatment (Figure 1E). The protein expression of RUNX2 and OCN showed a similar trend (Figures 1F,G). Interestingly, 10 μM naringenin showed the highest ability to promote osteogenic differentiation, showed by ARS staining, ALP activity and osteogenic marker expression, indicating the best dose of naringenin for osteogenesis was around 10 μM.

### Naringenin Inhibits Osteoclastogenic Differentiation of Bone Marrow Monocytes

**FIGURE 2:**
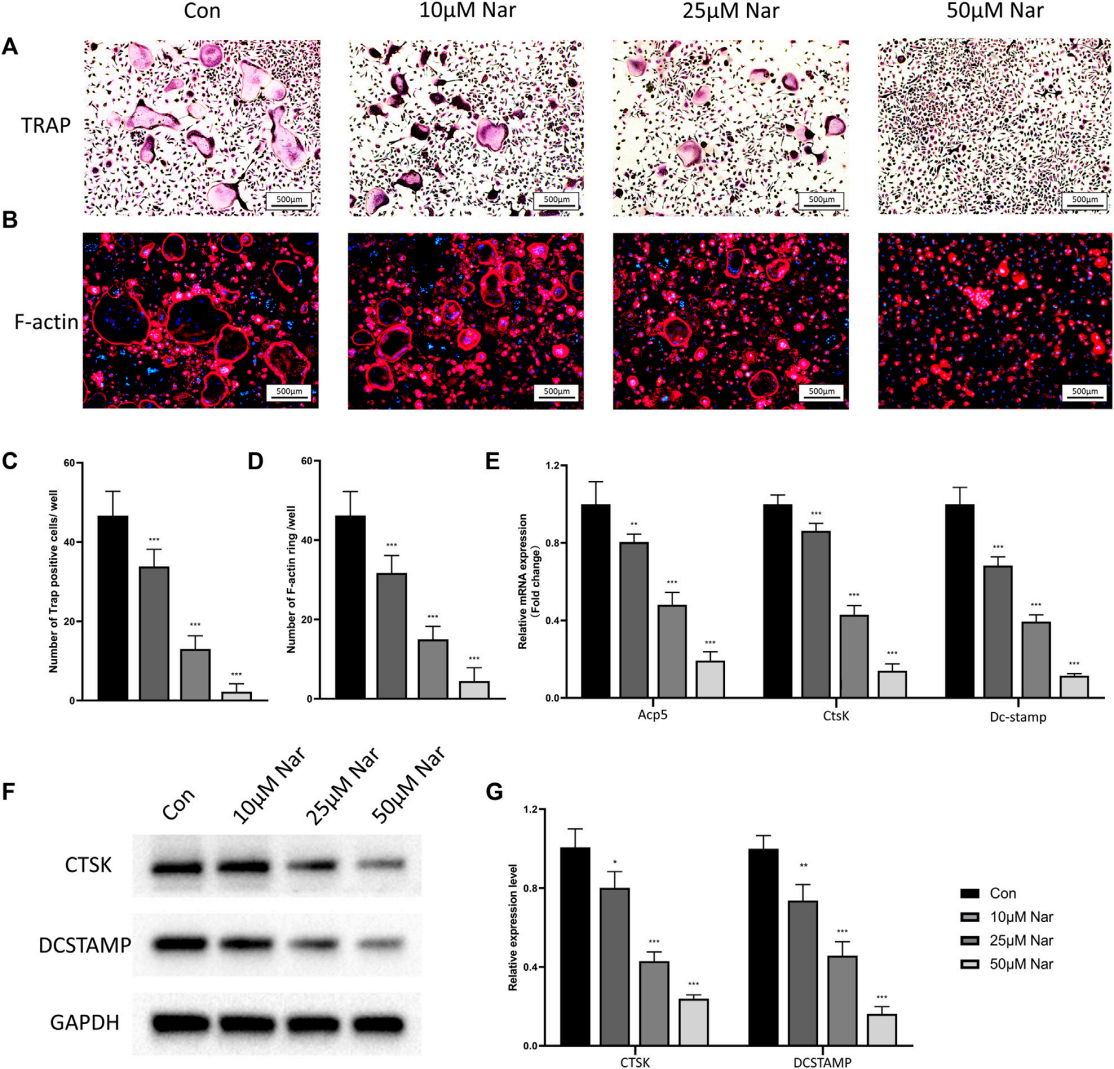
Naringenin inhibits osteoclastogenic differentiation of BMMs. **(A,C)** Formation of TRAP-positive cells and its quantification. **(B,D)** Formation of F-actin rings and its quantification. **(E)** Transcript levels of osteoclast markers acid phosphatase 5, tartrate resistant (Acp5), cathepsin K (CtsK) and dendritic cell-specific transmembrane protein (Dc-stamp). **(F,G)** Western blot and optical density analysis of CTSK and DCSTAMP with GAPDH as reference. *n* = 4 per group. Data are expressed as mean ± SD. * *p* < 0.05; ** *p* < 0.01; *** *p* < 0.001. * compared with con group.

Osteoclast differentiation was induced on primary BMMs *in vitro*. CCK-8 results showed 100 μM or less naringenin exerted no significant cytotoxic effects on BMMs, thus the doses of 10, 25, and 50 μM naringenin were chosen to treat the induced BMMs (Supplementary Figure S2B). As shown in Figures 2A,C, large TRAP + multinucleated osteoclasts were observed after 5 days of induction by M-CSF/RANKL. However, the formation of osteoclasts was significantly inhibited by naringenin in a dose dependent manner. The number of F-actin rings (the indicator of mature and functional osteoclast) also decreased dramatically after naringenin treatment (Figures 2B,D). RT-qPCR showed the transcriptional levels of osteoclastogenic markers including Acp5, CtsK, and Dc-stamp reduced notably (Figure 2E). Similarly, protein levels of CTSK and DCSTAMP were downregulated after naringenin treatment (Figures 2F,G). Taken together, osteoclast differentiation was suppressed by naringenin in a concentration dependent manner.

### Naringenin Regulates Macrophage Phenotype

**FIGURE 3:**
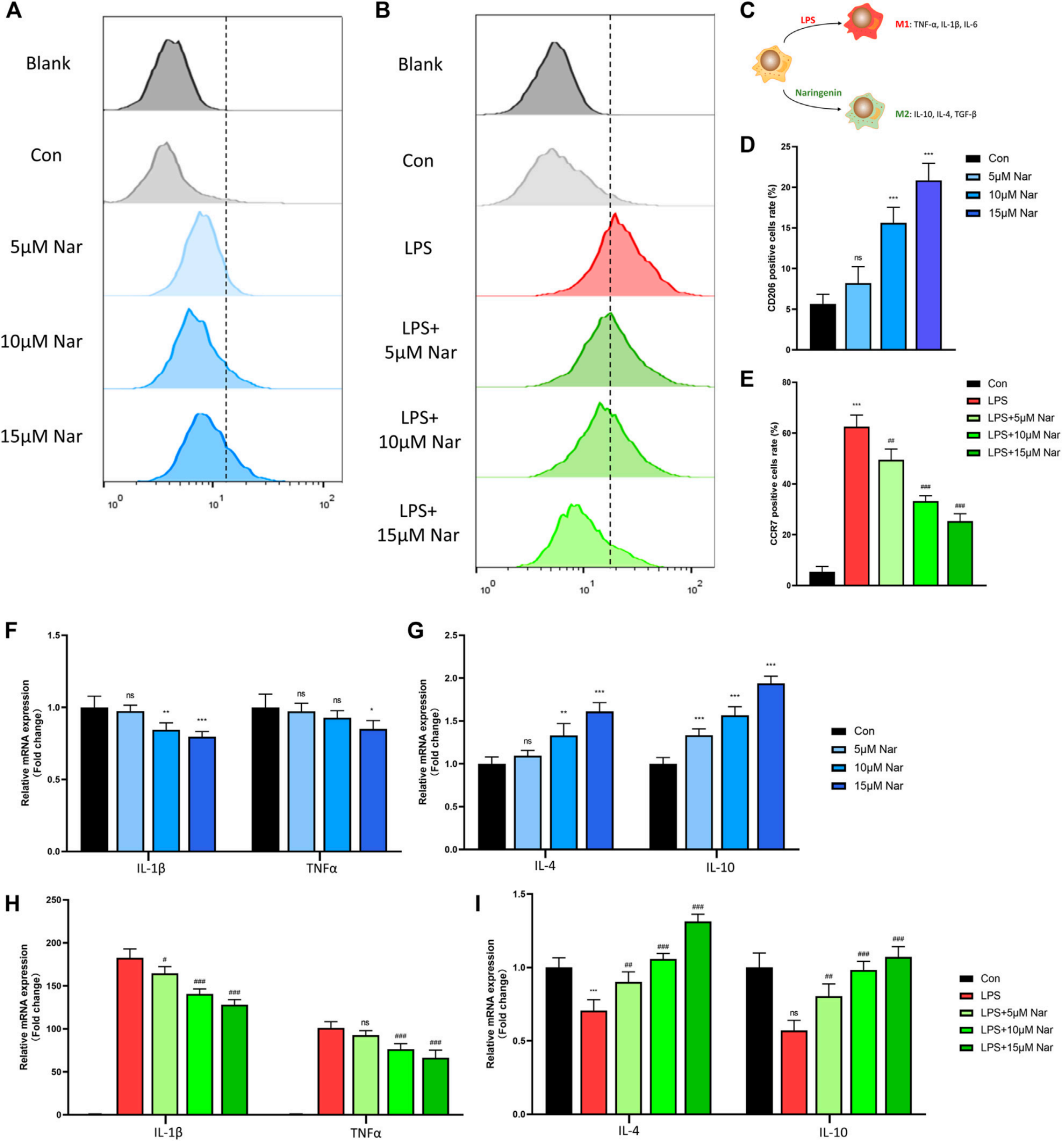
Naringenin regulates macrophages phenotype. **(A–E)** CCR7 and CD206 positive cells’ percentage of total cells analyzed by flow cytometry. **(C)** Schematic diagram of naringenin-mediated macrophage polarization. **(F-I)** Expressions of anti-inflammatory cytokines IL-4 and IL-10 as well as pro-inflammatory cytokines IL-1β and TNF-α in normal and inflammatory conditions. *n* = 4 per group. Data are expressed as mean ± SD. * *p* < 0.05; ** *p* < 0.01; *** *p* < 0.001;. * compared with con group.

Flow cytometry was performed to measure the rate of M1 macrophage (CCR7 positive) and M2 macrophage (CD206 positive) (Figure 3C). In non-inflammatory environment, the expression of CD206 were upregulated in a dose dependent manner (Figures 3A,D).Consistently, mRNA levels of CD206 and CD163, two M2 macrophage markers, were enhanced by naringenin treatment (Supplementary Figure S3A). However, due to the low expression of CCR7 of RAW264.7 cells in normal medium, no significant change in CCR7 expression was observed after naringenin treatment (data not shown). Thus, we use LPS to induce a proinflammatory environment. After LPS treatment, approximately 60% of cells expressed CCR7, suggesting the success in inducing a M1 phenotype (Figures 3B,E). RT-qPCR showed that with LPS incubation, the expressions of CCR7 and CD11c increased over 10-fold compared to con group (Supplementary Figure S3B). After LPS-treated RAW264.7 cells were cocultured with naringenin, the percentage of CCR7 positive cells were notably downregulated (Figures 3B,E). In accord, the transcriptional levels of CCR7 and CD11c declined by naringenin treatment in a concentration-dependent manner (Supplementary Figure S3B).

Then the levels of cytokines including IL-1β, TNF-α, IL-4, and IL-10 were measured by RT-qPCR. In non-inflammatory condition, the expression of IL-1β decreased significantly after 10 and 15 μM naringenin treatments (Figure 3F). In addition, TNFα level was downregulated only in 15 μM naringenin (Figure 3F). On the contrary, IL-4 and IL-10 increased after naringenin treatment in a concentration dependent pattern (Figure 3G). After the induction of LPS, the levels of TNFα and IL-1β increased over 100-fold compared to the control group (Figure 3H). With the treatment of naringenin, TNFα and IL-1β were significantly reduced especially in the concentration of 15μΜ (Figure 3H). Moreover, the expression of IL-4 and IL-10 decreased with LPS incubation (Figure 3I). A significant increase was observed after naringenin treatment (Figure 3I). According to that shown previously, naringenin showed great anti-inflammatory capacity and promoted M2-phenotype transition of macrophages.

### The Effects of Naringenin on the Expression of BMP-2 and TGF-β in Macrophages

**FIGURE 4:**
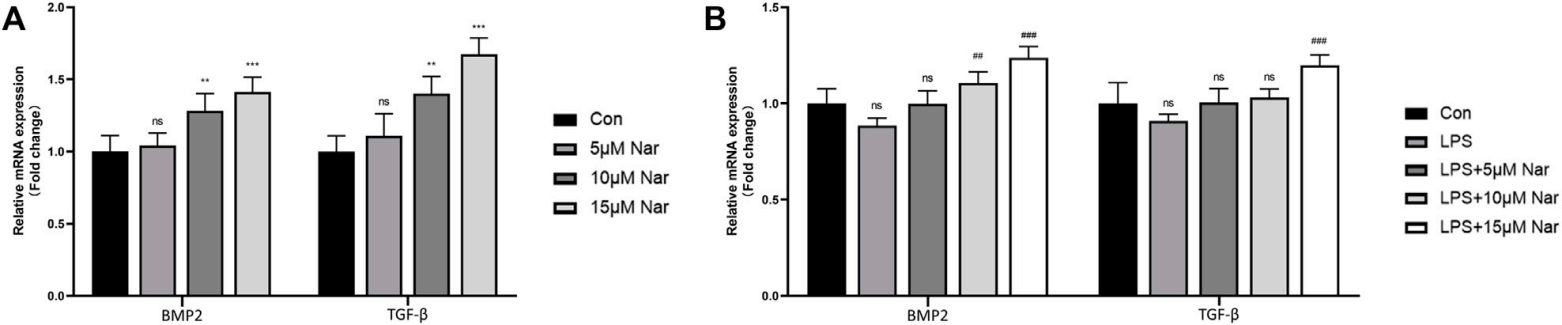
Effects of naringenin on the expression of BMP-2 and TGF-β in macrophages. The gene expressions of BMP-2 and TGF-β in response to naringenin under non-inflammatory environment **(A)** and inflammatory environment **(B)** measured by RT-qPCR. *n* = 4 per group. Data are expressed as mean ± SD. * # *p* < 0.05; ** ## *p* < 0.01; *** ### *p* < 0.001. * compared with control group; # compared with LPS group.

Previous studies demonstrated that BMP2 and TGFβ are two most important factors in the communication between BMSCs and macrophages (Pajarinen et al., 2019). Therefore, the alteration of these two molecules was measured by RT-qPCR. In normal cultured condition without LPS, BMP2 and TGF-β expression was significantly upregulated by 10 and 15 µM naringenin treatments, though no remarkable alteration in 5 µM naringenin coculture was observed in RAW264.7 cells (Figure 4A). After LPS exposure, the tendency of decrease in BMP2 and TGFβ was not significant (Figure 4B). However, 15 µM naringenin enhanced the levels of both BMP2 and TGFβ in LPS-treated condition (Figure 4B). These results suggested naringenin could promote the expression of BMP-2 and TGF-β, two osteogenic molecules, in macrophages derived from RAW264.7 cells.

### Osteogenesis is Enhanced Under a Naringenin-Modulated Macrophage Coculture Medium

**FIGURE 5:**
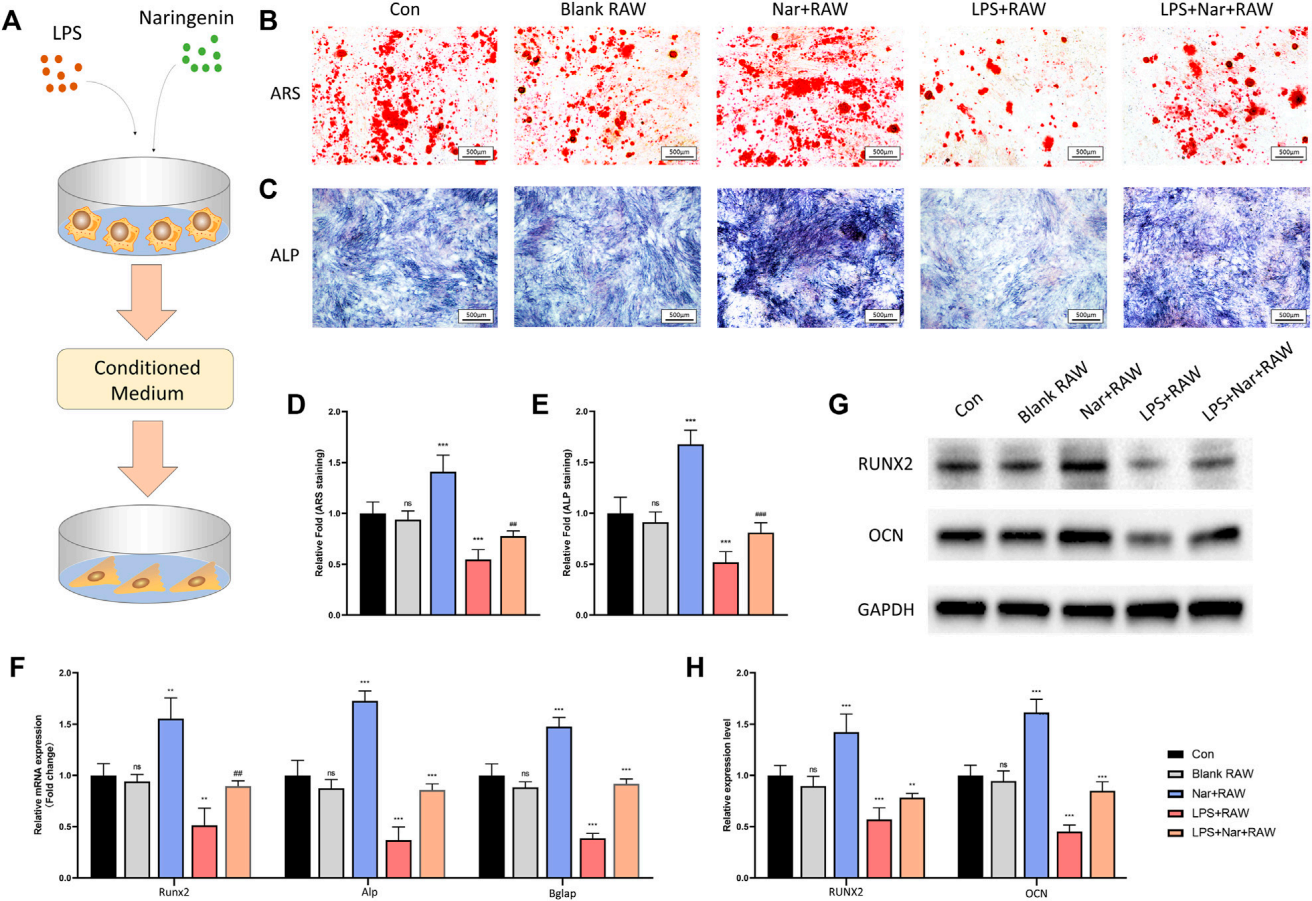
Osteogenesis is enhanced under naringenin-modulated macrophage coculture medium. **(A)** Schematic illustration. **(B,D)** ARS staining and its quantification. **(C,E)** ALP staining and its quantification. **(F)** Transcript levels of osteoclast markers Acp5, CtsK, and Dc-stamp. **(G, H)** Western blot and optical density analysis of RUNX2 and OCN with GAPDH as reference. *n* = 4 per group. Data are expressed as mean ± SD. * # *p* < 0.05; ** ## *p* < 0.01; *** ### *p* < 0.001. * compared with con group; # compared with LPS + RAW group.

To investigate the effects of naringenin/LPS treated macrophages on osteogenesis, RAW264.7 cells were cultured with or without naringenin/LPS for 24 h. Then the culture medium was collected and added into different groups of BMSCs for osteogenic differentiation. The schematic diagram of the cell processing method was shown in Figure 5A. The formation of calcium nodules shown by ARS staining and ALP activity had no significant change in normal RAW264.7 cell’s medium, but were notably enhanced after RAW264.7 cells were activated by naringenin (Figures 5B–E). On the contrary, medium from macrophages pretreated with LPS impeded osteoblastic differentiation of BMSCs manifested as diminished ARS positive quantity and ALP activity, which could be significantly reversed by naringenin coculture (Figures 5B–E). The mRNA levels of osteogenic marker genes including Runx2, Alp, and Bglap also increased in Nar + RAW group and decreased in LPS + RAW group compared to con group (Figure 5F). After naringenin coculture, the expression of Runx2, Alp, and Bglap was significant enhanced in Nar + LPS + RAW in comparison with LPS + RAW group (Figure 5F). A similar tendency of RUNX2 and OCN protein levels was observed (Figures 5G,H). Thus, we could conclude that naringenin-treated macrophages exhibited osteogenic property in both inflammatory and non-inflammatory conditions.

### Osteoclastogenesis is Suppressed Under a Naringenin-Modulated Macrophage Coculture Medium

**FIGURE 6:**
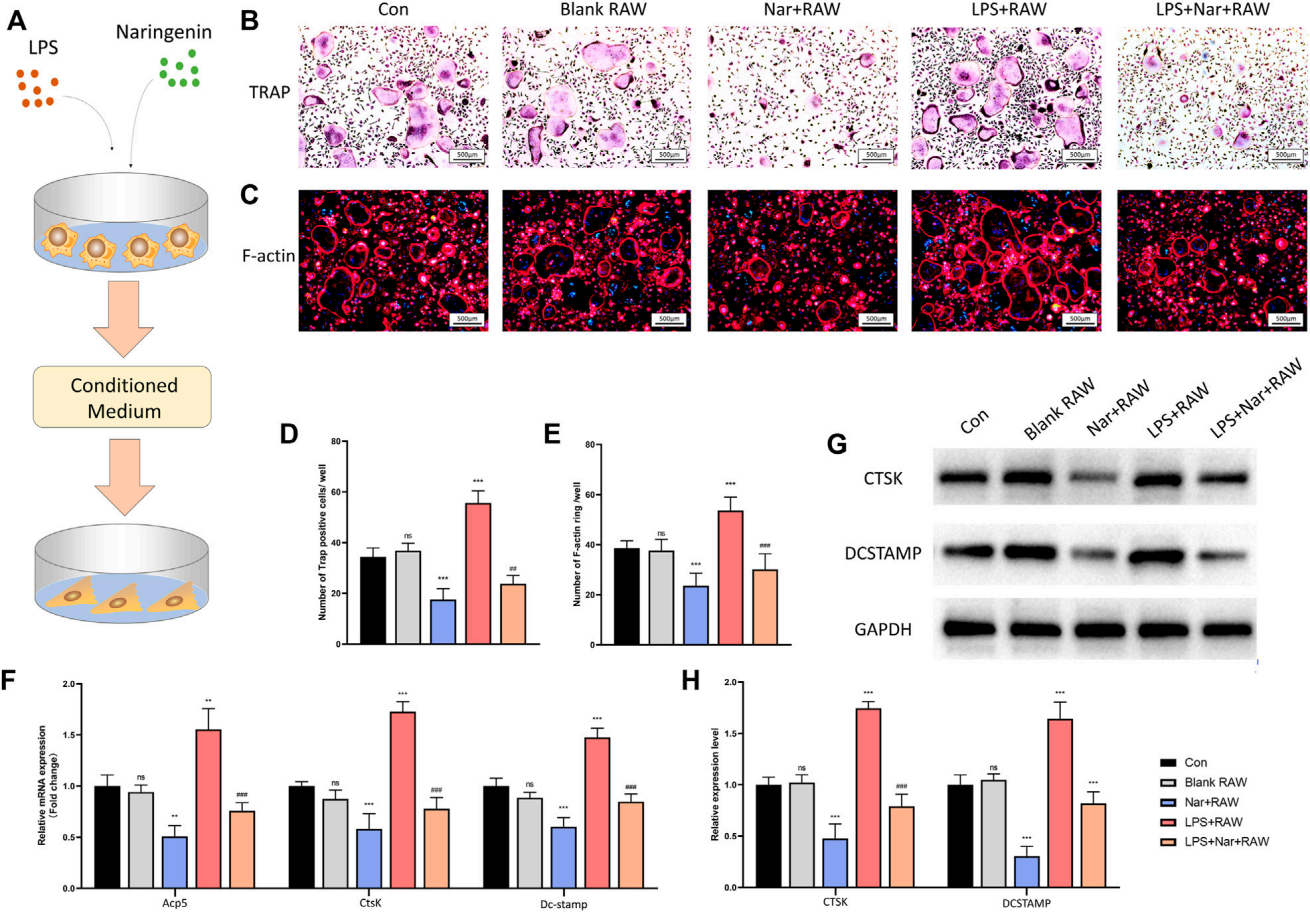
Osteoclastogenesis is suppressed under naringenin-modulated macrophage coculture medium. **(A)** Schematic illustration. **(B,D)** Formation of TRAP-positive cells and its quantification. **(C,E)** Formation of F-actin rings and its quantification. **(F)** Transcript levels of osteoblast markers Acp5, CtsK, and Dc-stamp. **(G, H)** Western blot and optical density analysis of CTSK and DCSTAMP with GAPDH as reference. *n* = 4 per group. Data are expressed as mean ± SD. * # *p* < 0.05; ** ## *p* < 0.01; *** ### *p* < 0.001. * compared with con group; # compared with LPS + RAW group.

To explore the influence of macrophages at different conditions on osteoclastogenesis, the supernatants of RAW264.7 cells treated with or without naringenin/LPS were collected and used in the osteoclast differentiation of BMMs (Figure 6A). TRAP staining and F-actin staining showed no significant alteration between blank RAW and con group (Figures 6B–E). The number of TRAP-positive cells and F-actin rings were significantly downregulated in the medium of RAW264.7 cells incubated with naringenin while they were upregulated by the medium with LPS (Figures 6B–E). In addition, after RAW264.7 cells were treated with both naringenin and LPS, the medium had less ability to promote osteoclastogenesis than the group incubated with LPS alone (Figures 6B–E). In accord, the transcriptional levels of Acp5, CtsK, and Dc-stamp, and translational levels of CTSK and DCSTAMP decreased in Nar + RAW group and increased in LPS + RAW group in comparison with con group (Figures 6F–H). The enhancement in LPS + RAW group could be alleviated after naringenin treatment (Figures 6F–H). These results showed that macrophages treated with naringenin inhibited osteoclast differentiation in normal condition and alleviated LPS induced excessive osteoclastogenesis.

### Naringenin Alleviates LPS-Induced Bone Loss

**FIGURE 7:**
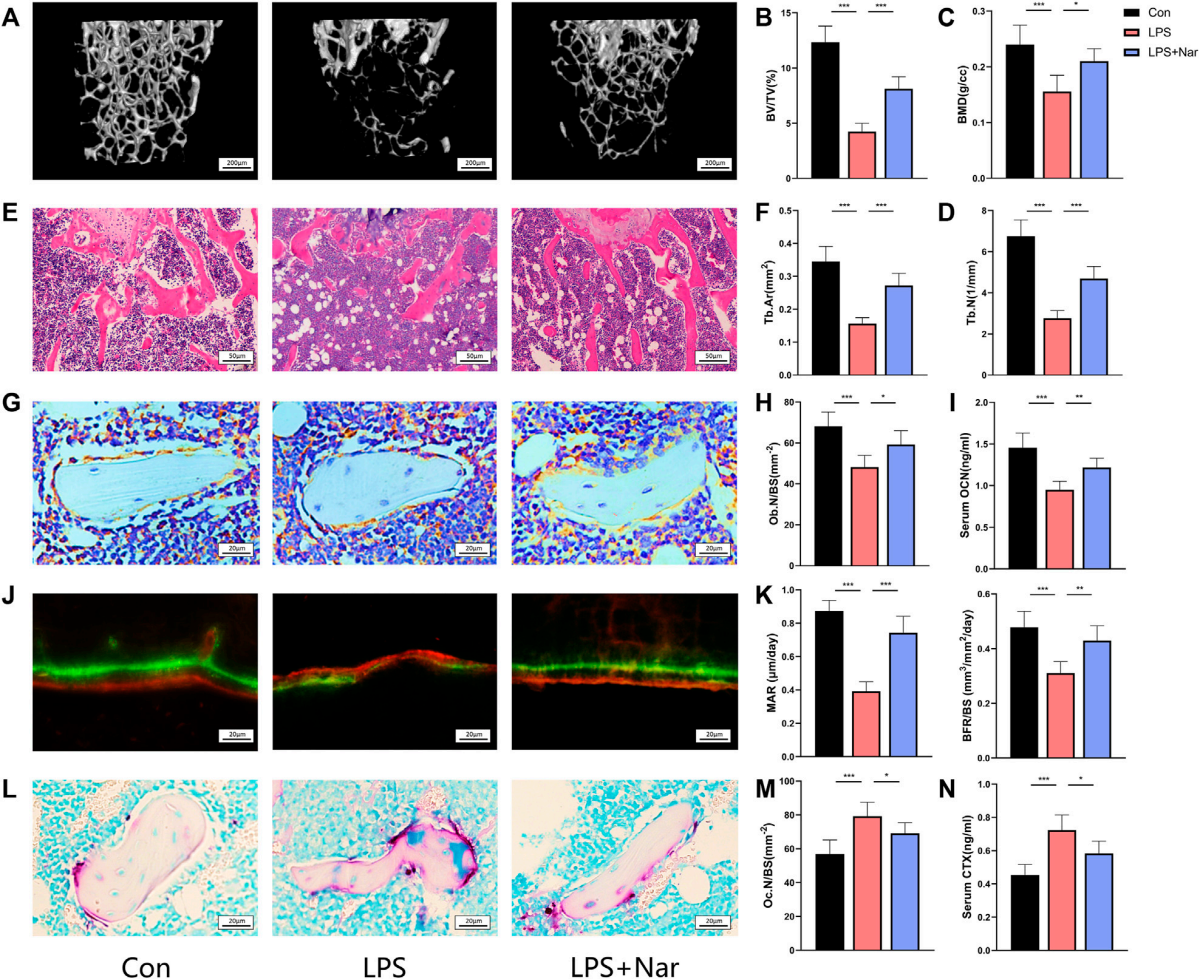
Naringenin alleviates LPS-induced bone loss. **(A)** Representative μCT analysis of trabecular bone of the distal femur. **(B–D)** Calculations of bone volume/total volume (BV/TV), trabecular bone mineral density (BMD), and trabecular number (Tb.N). **(E)** Representative H&E staining of distal femoral sections. **(F)** Quantification of trabecular area (Tb.Ar) from H&E staining. **(G)** Osteocalcin (OCN) stained sections of the distal femur. Original magnification ×40. **(H)** Osteoblast (OCN positive) number per bone surface (Ob.N/BS). **(I)** Serum level of osteoblast marker OCN. **(J)** Representative calcein staining of trabecula of distal femoral sections. **(K)** Quantification analysis of calcein staining. Mineral apposition rate (MAR) and bone formation rate per unit of bone surface (BFR/BS). **(L)** Tartrate-resistant acid phosphatase (TRAP) stained sections of the distal femur. Scale bar: 20 μm. **(M)** Osteoclast (nuclei>3; TRAP positive) number per bone surface (Ob.N/BS). **(N)** Serum level of osteoclast marker CTX-I. *n* = 5 replicates per group in all panels. Data are expressed as mean ± SD. * *p* < 0.05; ** *p* < 0.01; *** *p* < 0.001.

To further confirm the effects on bone metabolism, naringenin was used to treat LPS-induced inflammatory bone loss on mice. After euthanizing the mice, the blood and femora were collected and analyzed. Trabecular BV/TV and BMD significantly decreased after LPS injection (Figures 7A–C). In addition, bone microstructure also deteriorated which was indicated by the reduction of Tb.N and Tb.Th with the increase of Tb.Sp (Figures 7A,D; Supplementary Figures S4A,B). Naringenin treatment significantly attenuates the LPS-induced bone loss and bone-microarchitecture destruction (Figures 7A–D). However, cortical thickness and area did not differ among all three groups (Supplementary Figures S4C–E). In accord, HE staining also showed the decrease of Tb.Ar caused by LPS was notably reversed by naringenin treatment (Figures 7E,F). To further investigate the change of bone remodeling, osteocalcin immunohistochemistry, calcein staining, TRAP staining were performed to evaluate the number and function of osteoblasts/osteoclasts. The quantity of Ocn positive cells (osteoblasts) decreased significantly after LPS administration (Figures 7G,H). Mineral apposition rate (MAR) and bone formation rate (BFR) were also reduced in LPS group, indicating LPS injection led to impaired bone formation (Figures 7J,K). In accord, serum OCN level also declined in LPS-treated mice (Figure 7I). Naringenin treatment significantly alleviated the exacerbation of bone formation, shown by increased OCN expressing cells, improved MAR and BFR, and enhanced OCN level (Figures 7G–K). In addition, TRAP-positive cells with serum CTX-1 level (serum marker for osteoclast function) increased in LPS group, and were attenuated by naringenin administration (Figures 7L–N). According to that shown previously, the LPS-induced imbalance in bone remodeling by the suppression of bone formation and the promotion of bone resorption was significantly alleviated by naringenin treatment.

### Naringenin Promotes the Healing of Calvarial Defect

**FIGURE 8:**
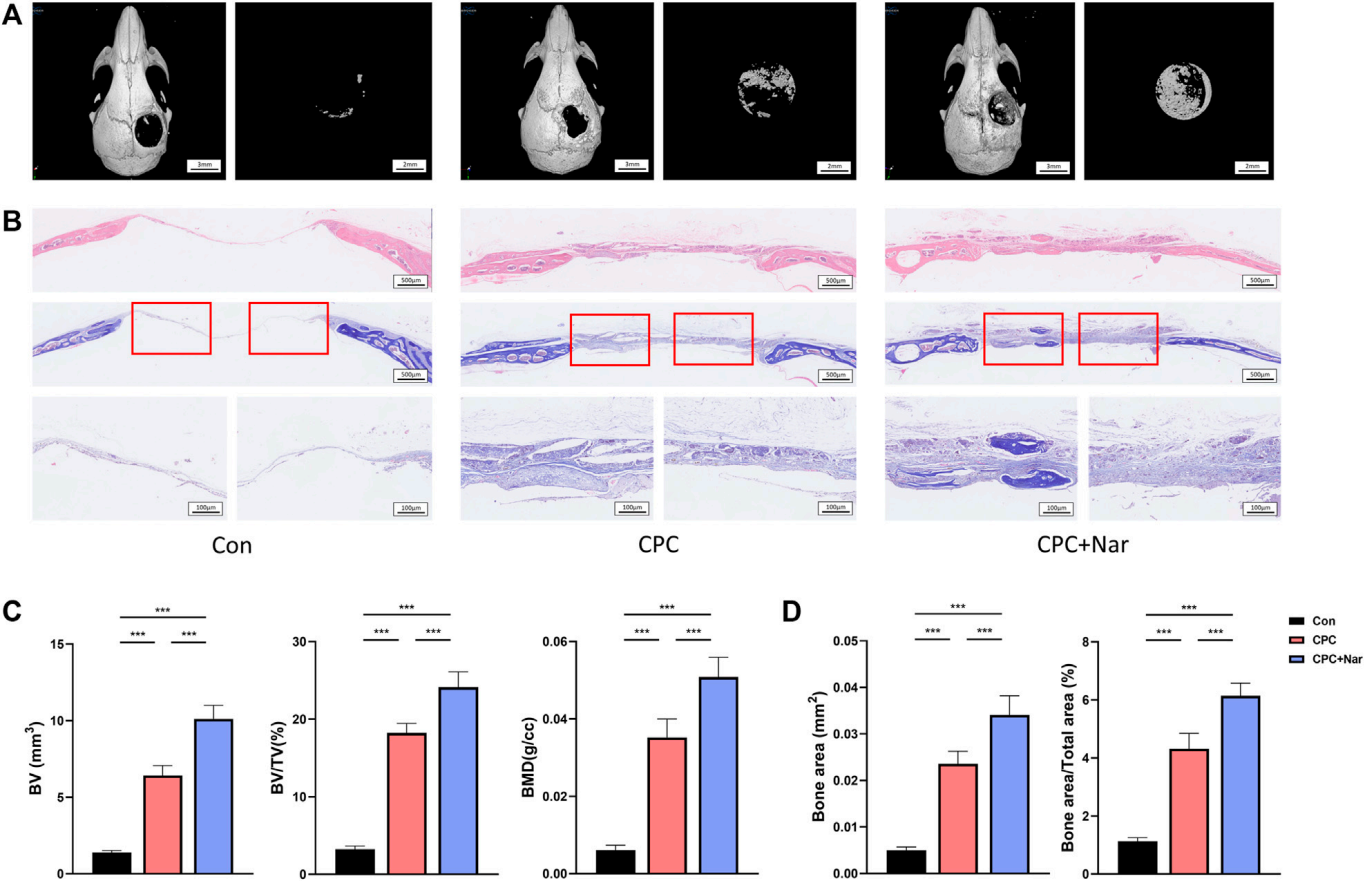
Naringenin promotes the healing of calvarial defect. **(A)** Representative μCT analysis of the calvarial defects. **(B)** HE staining and Masson staining of the calvarial defects. **(C)** Calculations of bone volume (BV), BV/TV, and BMD of new bone in the calvarial defects. **(D)** Quantification of bone area and bone area/total area of new bone in the calvarial defects from Masson staining. *n* = 5 replicates per group in all panels. Data are expressed as mean ± SD. * *p* < 0.05; ** *p* < 0.01; *** *p* < 0.001.

To investigate if naringenin promotes bone regeneration, a calvarial bone-defect model was constructed in mice. The defect was either left with nothing or filled with calcium phosphate cement (CPC). Also, naringenin was injected intraperitoneally as a supplement for bone formation after the CPC scaffold was implanted. Four weeks after the bone defect was created, μCT showed a great increase of new bone (manifested as BV, BV/TV, and BMD) in CPC group than con group (Figures 8A,C). In addition, the administration of naringenin significantly reinforced the new bone formation by CPC filling (Figures 8A,C). HE staining and Masson staining were also performed. In accord, naringenin significantly enhanced the new bone area after CPC scaffold was implanted (Figures 8B,D). These results suggest that naringenin promotes bone formation and is a potential treatment for fracture or bone defect.

## Discussion

In this study, we demonstrated that naringenin could promote osteogenesis and suppress osteoclastogenesis directly. In addition, naringenin showed great ability to induce M2-phenotype transition of macrophages in both inflammatory and non-inflammatory environment *in vitro*. Coculture with naringenin-treated macrophage medium significantly enhanced osteogenic capacity and inhibited osteoclastic potential of primary bone marrow cells. *In vivo* experiments also showed that naringenin treatment alleviated LPS-induced bone loss and assisted bone regeneration in calvarial defect model.

Naringenin has been recognized as a modulator of immune system and bone metabolism (Bhia et al., 2021). Several studies emphasized naringenin inhibits NF-κB and MAPK activation in different cell types such as adipocytes, hepatocytes, cancer cells, and macrophages to exert anti-inflammatory and antioxidant effects on acute and chronic inflammation including colitis, arthritis, gastric cancer, and obesity (Zeng et al., 2018). In addition, naringenin improves osteogenic differentiation of rat BMSCs and hPDLSCs through SDF-1/CXCR4 signaling pathway (Zhang L. et al., 2021; Wang et al., 2021). Wang et al. reported that naringenin impedes osteoclast differentiation by the inhibition of p38 signaling and enhancing helper T cells-secreted IL-4 (Wang et al., 2014; Wang et al., 2018). In this study, we confirmed naringenin treatment promoted osteoblastic differentiation of mice BMSCs *in vitro* and the best concentration was around 10–15 μM. RANKL induced osteoclastogenesis was inhibited by naringenin in a dose dependent manner and 50 μM showed a complete suppression. We also found that 15 μM naringenin was able to promote a M2 transition in non-inflammatory condition and significantly suppress LPS-induced M1 polarization of macrophages. These results encouraged us to further explore the modulation of naringenin on the crosstalk between macrophage and bone remodeling.

Macrophages are essential in the regulation of bone formation and resorption (Muñoz et al., 2020). A special population of resident macrophages (osteomacs) was found distributed throughout the endosteum and periosteum, which forms canopy-like structure around osteoblasts (Burnett et al., 2004; Chang et al., 2008). *In vivo* depletion of macrophages by a macrophage-fas-induced apoptosis (MAFIA) model leads to the loss of active bone-forming surface, suggesting macrophages are vital for osteoblast survival and function (Burnett et al., 2004). On the other hand, macrophages and osteoclasts share the origin from hematopoietic stem cells and part of macrophages could differentiate into osteoclasts at the stimulation of M-CSF and RANKL, indicating the close relationship between these 2 cell types (Lee et al., 2021). In addition, lots of studies revealed that polarized macrophages serve as important constituent of immune environment for bone homeostasis. In general, M1 macrophages were found to be associated with bone destruction and M2 macrophages with bone construction, in which macrophages secreted cytokines plays an important role (Schlundt et al., 2021). Proinflammatory factors such as TNF-α and IL-1β derived from M1 phenotype macrophages inhibit the expression of osteogenic genes including Runx2 and Alp in BMSCs, while significantly enhance osteoclastogenesis by NF-κB and MAPK activation (Chen et al., 2021). On the contrary, IL-4 and IL-10 released from M2 macrophages impede osteoclast differentiation (von Kaeppler et al., 2021; Wallimann et al., 2021). Moreover, M2 macrophages secrete BMP2 and TGF-β as facilitators for osteogenesis (Horwood, 2016). In this study, we found that naringenin enhanced IL-4, IL-10, BMP2, and TGF-β levels in macrophages in both inflammatory and non-inflammatory environment. LPS induced significant increase in M1 macrophage-derived proinflammatory TNF-α and IL-1β, which was reversed by naringenin treatment. Then, we established a coculture system by performing the osteoblastic and osteoclastic differentiation assay in macrophage conditioned medium, which preserved soluble factors without direct cell contact. In normal condition, the medium of 15 μM naringenin-treated RAW264.7 significantly enhanced the ARS staining, ALP activity and osteogenic markers expression in BMSCs, while inhibited mature osteoclast formation. On the contrary, coculture with LPS-incubated RAW264.7 medium notably hampered osteogenic differentiation and elevated osteoclast number, which could be alleviated by naringenin treatment. These data demonstrated that naringenin could modulate the interactions between macrophages and osteoblasts/osteoclasts.

To further confirm the therapeutic potential of naringenin on inflammation related osteopenia, mice model of LPS-induced bone loss was built to assess the naringenin’s effects *in vivo*. In accord with previous reports (Place et al., 2021), long-term LPS injection caused bone mass decrease and architecture destruction, of which the cellular basis is the impairment of osteoblast function and excessive osteoclast activity. Naringenin administration remarkably alleviated LPS-induced bone loss *via* restoring the balance between osteoblasts and osteoclasts. In addition, considering the important role of macrophages in the healing of bone fracture, naringenin was used to treat mice with critical sized calvarial defect. Our data showed there was little new bone formation in defects in the natural condition. After CPC, a clinically used bone cement, was filled into the defect, obvious new bone formation was observed, which was significantly increased by naringenin treatment. According to that shown previously, these data showed naringenin exerts anabolic effects *in vivo*.

Our results confirmed that naringenin promotes osteogenic differentiation and inhibits osteoclastogenic differentiation directly. Also, for the first time, we found that naringenin could improve immune microenvironment *via* modulating macrophage polarization and cytokines secretion to rebalance the bone formation and bone resorption. Its capacity of attenuating inflammatory osteopenia and promoting bone regeneration was also confirmed by *in vivo* experiments. These findings demonstrated that naringenin is a potential therapeutic agent for pathological bone loss.

## Data Availability

The original contributions presented in the study are included in the article/Supplementary Materials, further inquiries can be directed to the corresponding authors.

## Abbreviations

Acp5, acid phosphatase 5, tartrate resistant; Alp, alkaline phosphatase; AMPK, AMP-activated protein kinase; ATF3, activating transcription factor 3; ARS, Alizarin Red S; ALP, alkaline phosphatase; BFR, bone formation rate; Bglap, bone gamma-carboxyglutamate protein; BMP2, bone morphogenetic protein 2; CCR7, C-C motif chemokine receptor 7; (CD86, CD163, CD206), cluster of differentiation 86, 163, 206; CCK-8, cell counting kit-8; CtsK, cathepsin K; CTX-1, type I C-terminal telopeptide; CPC, calcium phosphate cement; CXCR-4, C-X-C chemokine receptor type 4; DC-STAMP, dendritic cell-specific transmembrane protein; hPDLSCs, human periodontal ligament stem cells; (IL-1β, IL-4, IL-10), interleukin-1 beta, 4, 10;MAFIA, macrophage-fas-induced apoptosis; MAPK, mitogen-activated protein kinase; MAR, mineral apposition rate; M-CSF, macrophage colony-stimulating factor; NF-κB, nuclear factor kappa B; RANKL, receptor activator of nuclear factor-κB ligand; RT-qPCR, quantitative real-time polymerase chain reaction; Runx2, Runt-related transcription factor 2; SDF-1, stromal cell-derived factor-1; TGF-β, transforming growth factor beta.
