# Supplementary material for: Naringenin is a Potential Anabolic Treatment for Bone Loss by Modulating Osteogenesis, Osteoclastogenesis, and Macrophage Polarization
Source: Front Pharmacol. 2022 May 2;13:872188. doi: 10.3389/fphar.2022.872188 (PMC9108355; doi:10.3389/fphar.2022.872188)
Supplement: Supplementary file 1 [file DataSheet1.docx]

Table S1. Sequences of the primers used in this study.

| Gene Name | Primers |
| --- | --- |
| Runx2 | Forward 5’- GTGGTAGGCAGTCCCACTTT-3’ |
|  | Reverse 5’- AAAGTTTGCACCGCACTTGT-3’ |
| Alp | Forward 5’- CCCACCCAATTGCAGAGACT-3’ |
|  | Reverse 5’- ACCCCGCTATTCCAAACAGG-3’ |
| Bglap | Forward 5’- GCAGAACAGACAAGTCCCACAC-3’ |
|  | Reverse 5’- GTCAGCAGAGTGAGCAGAAAGAT-3’ |
| Acp5 | Forward 5’- ATGGGCGCTGACTTCATCAT -3’ |
|  | Reverse 5’- GGTCTCCTGGAACCTCTTGT -3’ |
| CtsK | Forward 5’- AGTAGCCACGCTTCCTATCC -3’ |
|  | Reverse 5’- CCATGGGTAGCAGCAGAAAC -3’ |
| Dc-stamp | Forward 5’- ACAAACAGTTCCAAAGCTTGC -3’ |
|  | Reverse 5’- TCCTTGGGTTCCTTGCTTC -3’ |
| CCR7 | Forward 5’- TGTACGAGTCGGTGTGCTTC -3’ |
|  | Reverse 5’- GGTAGGTATCCGTCATGGTCTTG -3’ |
| CD11c | Forward 5’- CAAGAAGCACCGAACATGGTT -3’ |
|  | Reverse 5’- GTCTGAGCTAGAGTCACTGGT -3’ |
| CD206 | Forward 5’- CTCTGTTCAGCTATTGGACGC -3’ |
|  | Reverse 5’- CGGAATTTCTGGGATTCAGCTTC -3’ |
| CD163 | Forward 5’- CTGGCCTCTGAGTTTAGGGTC -3’ |
|  | Reverse 5’- CCCTTGGTGTCGAACCAGC -3’ |
| IL-1β | Forward 5’- TTCAGGCAGGCAGTATCACTC-3’ |
|  | Reverse 5’- GAAGGTCCACGGGAAAGACAC-3’ |
| TNFα | Forward 5’- CCCTCACACTCAGATCATCTTCT-3’ |
|  | Reverse 5’- GCTACGACGTGGGCTACAG-3’ |
| IL-4 | Forward 5’- GGTCTCAACCCCCAGCTAGT-3’ |
|  | Reverse 5’- GCCGATGATCTCTCTCAAGTGAT-3’ |
| IL-10 | Forward 5’- GCTCTTACTGACTGGCATGAG-3’ |
|  | Reverse 5’- CGCAGCTCTAGGAGCATGTG-3’ |
| BMP2 | Forward 5’- GGGACCCGCTGTCTTCTAGT-3’ |
|  | Reverse 5’- TCAACTCAAATTCGCTGAGGAC-3’ |
| TGF-β | Forward 5’- CCACCTGCAAGACCATCGAC-3’ |
|  | Reverse 5’- CTGGCGAGCCTTAGTTTGGAC-3’ |
| GAPDH | Forward 5’- AGGTGGTGAAGCAGGCATCTGA -3’ |
|  | Reverse 5’- CGGCATCGAAGGTGGAAGAGTG -3’ |


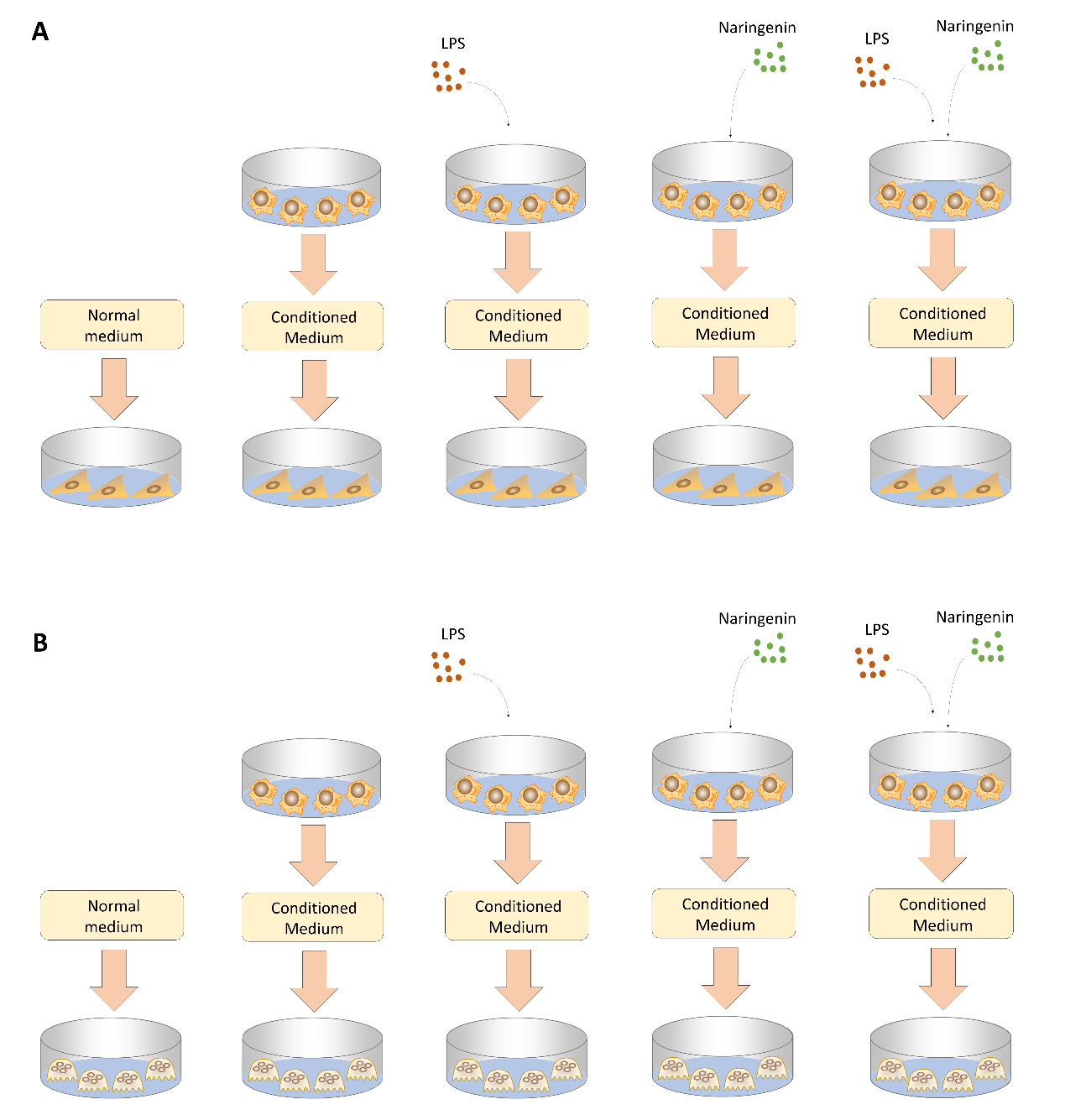


Figure S1. Schematic illustration for the co-culture of macrophages derived from RAW264.7 cells with BMSCs (A) and BMMs (B).


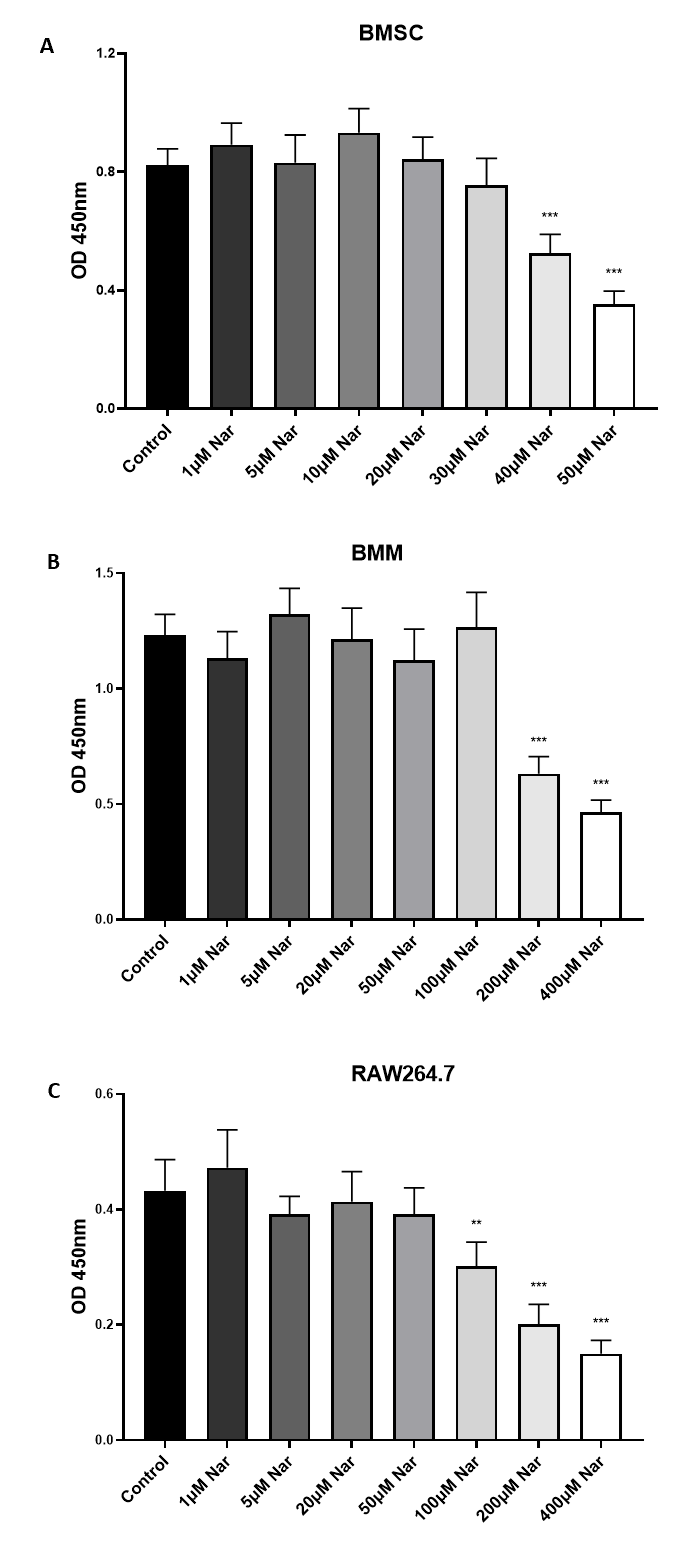


Figure S2. Cytotoxic effects of naringenin measured by CCK-8. (A-C) Optical density value of BMSCs (A), BMMs (B) and RAW264.7 (C) treated with naringenin in different concentrations. n = 4 per group. Data are expressed as mean ± SD. * p < 0.05; ** p < 0.01; *** p < 0.001. * compared with Control group.


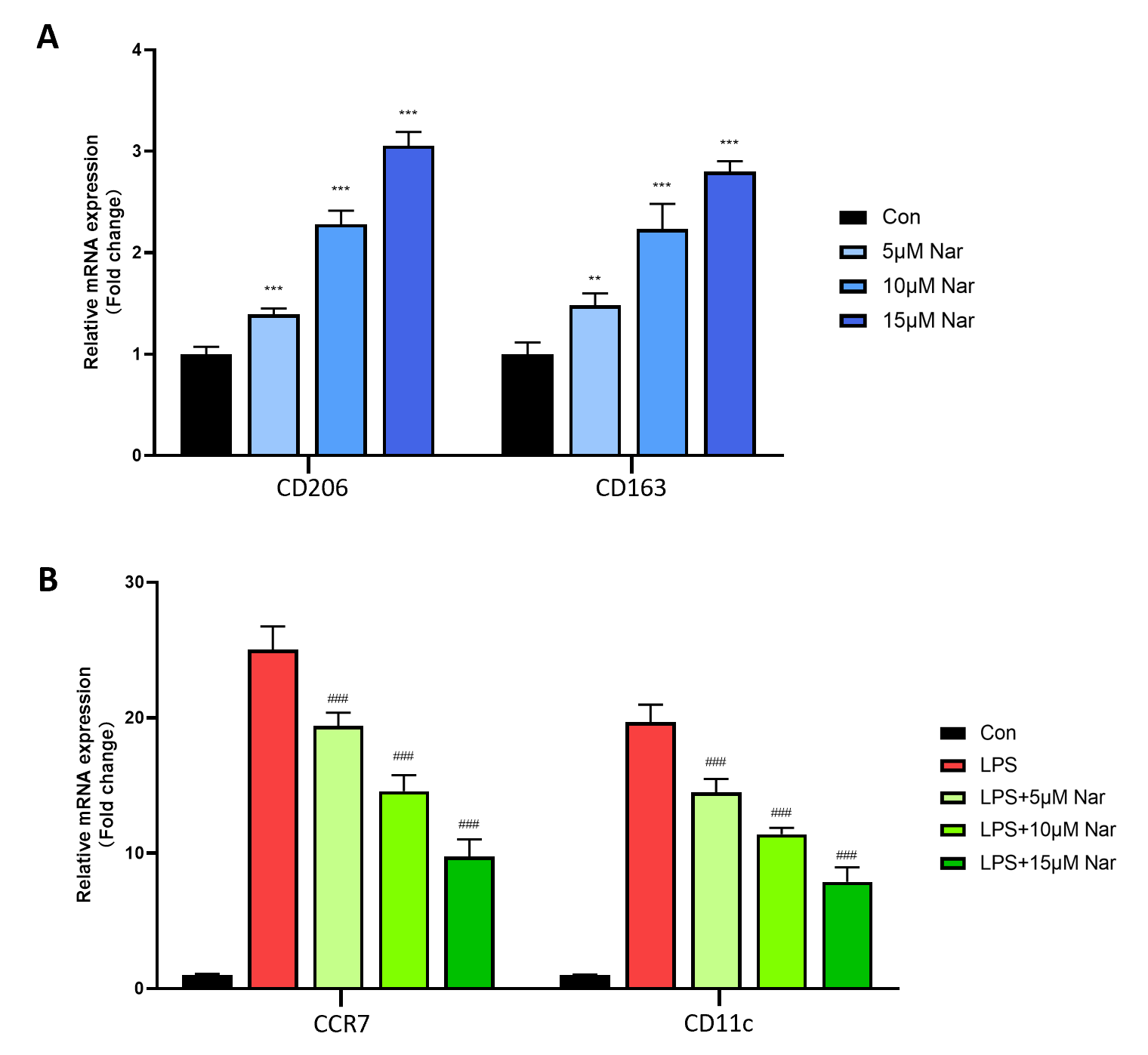


Figure S3. Expressions of CD206 and CD163 (M2 macrophage markers), along with CCR7 and CD11c (M1 macrophage markers) evaluated by RT-qPCR. n = 4 per group. Data are expressed as mean ± SD. * # *p* < 0.05; ** ## *p* < 0.01; *** ### *p* < 0.001. * compared with Control group; # compared with LPS group.


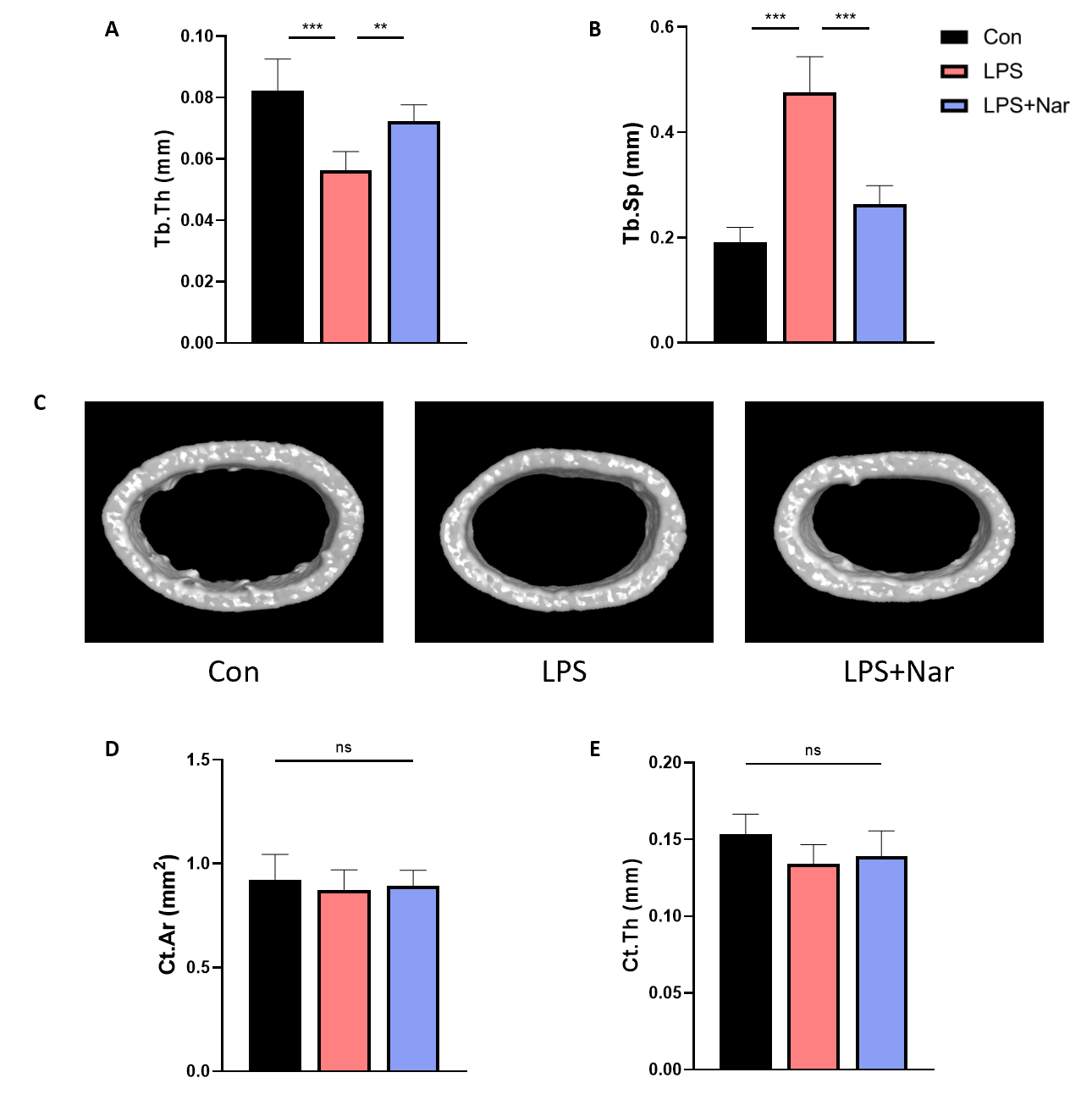


Figure S4. Naringenin alleviates LPS induced bone loss. (A) Calculations of trabecular thickness (Tb.Th) and Trabecular space (Tb.Sp) from μCT. (B) Representative μCT analysis of cortical bone of the distal femur. (D, E) Calculations of cortical area (Cr.Ar) and cortical thickness (Cr.Th) from μCT. n = 5 per group. Data are expressed as mean ± SD. * *p* < 0.05; ** *p* < 0.01; *** *p* < 0.001.
